# Ecosystem engineering creates a direct nutritional link between 600-m deep cold-water coral mounds and surface productivity

**DOI:** 10.1038/srep35057

**Published:** 2016-10-11

**Authors:** Karline Soetaert, Christian Mohn, Anna Rengstorf, Anthony Grehan, Dick van Oevelen

**Affiliations:** 1 NIOZ Royal Netherlands Institute for Sea Research, Department of Estuarine and Delta Systems, and Utrecht University, Yerseke, The Netherlands; 2 University of Aarhus, Department of Bioscience, Denmark; 3 University of Galway, Earth and Ocean Sciences, Ireland

## Abstract

Cold-water corals (CWCs) form large mounds on the seafloor that are hotspots of biodiversity in the deep sea, but it remains enigmatic how CWCs can thrive in this food-limited environment. Here, we infer from model simulations that the interaction between tidal currents and CWC-formed mounds induces downwelling events of surface water that brings organic matter to 600-m deep CWCs. This positive feedback between CWC growth on carbonate mounds and enhanced food supply is essential for their sustenance in the deep sea and represents an example of ecosystem engineering of unparalleled magnitude. This ’topographically-enhanced carbon pump’ leaks organic matter that settles at greater depths. The ubiquitous presence of biogenic and geological topographies along ocean margins suggests that carbon sequestration through this pump is of global importance. These results indicate that enhanced stratification and lower surface productivity, both expected consequences of climate change, may negatively impact the energy balance of CWCs.

Cold-water corals (CWC) are ecosystem engineers building extensive reefs that are habitat for a diverse fauna, such as sponges, crinoids and fish, making them hotspots of biodiversity^1^ and significant sinks of organic matter^2^,^3^ in the deep sea. Food limitation exerts a well-known control on biological activity in the deep sea, as the organic matter is produced in the sunlit surface ocean and rapidly degrades during the 100 s to 1,000 s of meters transit to the seafloor^4^. It is paradigmatic how diverse and productive CWCs can thrive in this resource-limited environment. A better understanding of how CWCs meet their nutritional demands is critically needed to assess how they will cope with climate-induced shifts in primary production^5^ and reduced water column mixing^6^. Moreover, it is increasingly clear that food availability is a key factor determining the sensitivity of CWCs to ocean acidification^7^,^8^.

Scleractinian CWCs form bottom topographies that are tens to hundreds meters high over thousands to millions of years^9^. The global distribution of CWCs renders carbonate mound and reef structures prominent topographic features of the seafloor along continental margins (Fig. 1). Recent studies in Scotland and Norway have suggested that the interaction of tidal currents with CWC reefs induces downwelling of surface water^10^,^11^,^12^ due to the formation of internal waves or hydraulic jumps^10^,^12^. As CWCs themselves form prominent topographies on the seafloor *and* these topographies induce downwelling of surface water, two intriguing questions naturally arise. Is there a positive feedback between CWC growth on carbonate mounds and food supply from the surface ocean? And, what are the implications for organic matter sequestration to the deep ocean?

In this study, we identify pathways of organic matter supply to 600-m deep CWCs along Rockall Bank (NE Atlantic) and provide evidence for a positive feedback between organic matter supply and CWC growth. Rockall Bank is rich in CWCs and represents a perfect study arena, because strongly contrasting CWC growth forms are found here (Fig. 2). On the eastern part, CWCs are predominantly found on the shelf-break ridge at 400 to 500 m water depth^13^,^14^ (Fig. 2). Just tens of kilometres to the west, the Logachev Mounds are found, a conspicuous CWC mound cluster of several kilometres long and up to 380 m high at a water depth of 600 to 1000 m 15,16. A coupled model was used to infer the organic matter supply pathways for the two contrasting CWC-rich regions at Rockall Bank (Methods). A 3D-hydrodynamic model^17^ is used to identify the frequency and intensity of downwelling events above the CWC mounds and the shelf break ridge. These hydrodynamic simulations were used to model organic matter dynamics in the water column. CWC uptake of suspended organic matter was imposed by combining their metabolic requirements^2^,^3^ with their occurrences based on a regional habitat suitability model^14^. We also quantitatively determine how the presence of CWC mounds influences organic carbon sequestration in the deep sea. The results of our modelling approach will be presented first, followed by the implications of our findings.

## Results and Discussion

### Cold-water coral mounds induce up- and downwelling events

A 3D-hydrodynamic model generated flow fields along the eastern Rockall Bank (NE Atlantic Ocean) shelf break^17^ (Methods). The model domain covers an area (60 × 90 km) of Rockall Bank and extends from the shallow bank (200 m) down into the deep Rockall Trough (2000 m) (Fig. 2). We focus on two depth-transects within this model domain, one superimposed on the shelf-break ridge and another on the Logachev mound cluster (Fig. 2).

The monthly-averaged residual currents, calculated from the hydrodynamic model output, show that surface water enters the region primarily from the southeast, roughly perpendicular to the continental slope, and deflects southwest to Logachev mounds and northeast along the shelf break (Supplementary Figure 1). The residual near-bottom currents are heterogeneous and substantially stronger (up to >0.4 m s^−1^ in the Logachev mound province and <0.25 m s^−1^ on the ridge) than the surface currents and are directed southwest (Supplementary Figure 1).

Superimposed on these long-term residual currents however, are localised up- and downwelling events that show up in time series of vertical velocities (Fig. 3A–D, Supplementary Movie 1). While the average vertical current speed is between 0.002 and 0.004 m s^−1^, the maximum vertical up- and downwelling currents in the water column above the Logachev mounds reach peak values of up to 0.15 and 0.28 m s^−1^, respectively (Fig. 4A), whereas vertical current velocities are substantially weaker above the CWC on the coral ridge (Fig. 4B). The frequency of these events is however comparable in both areas and are driven by the tidal cycle (Fig. 4).

Stratified tidal flow over finite-amplitude topography can generate a multitude of disturbances in the flow and water-column stratification, including internal waves and upwelling or downwelling^18^,^19^. The initial response of tidal flow over a sill or bank, such as the large mounds of the Logachev Mound cluster, is a stationary lee wave adding a vertical component to the stratified flow and a downstream depression of isopycnals^19^. At the turn of tide the flow slackens and the lee wave can propagate upstream as a non-linear internal tidal bore or true internal hydraulic jump^20^. Recent studies identified tidally driven non-linear hydraulic control of flow over an abrupt topography as the major driver for rapid downwelling and vertical mixing at shallow CWC reefs^10^,^12^ and within the Logachev Mound province^21^, which may intensify in areas of strong depth variations and during periods of strongly amplified flow such as the spring/neap tidal cycle (Fig. 3, refs 12 and 21).

### A topographically-enhanced organic matter pump

The flow fields of the hydrodynamic model are used to force a biogeochemical reaction-transport model, in which organic matter, produced in the photic zone, is subjected to passive sinking, hydrodynamical transport and biological degradation (see Methods). In the model, organic matter refers to a mixture of suspended sources (e.g. dissolved organic matter, bacteria, marine snow and small zooplankton) that are transported largely by hydrodynamics. *Lophelia pertusa*, together with other filter feeders in the reef community, is an opportunistic feeder that can feed on a range of organic matter sources^22^. Hence, this coupled hydrodynamic-biogeochemical model takes suspended organic matter as a non-conservative tracer to reconstruct organic food supply to the CWC-rich regions.

Model simulations reveal clearly contrasting transport mechanisms between both regions (Fig. 3 and the Supplementary Movie showing vertical current velocities and organic matter concentrations during the entire simulation period at 6 hour resolution).

The downwelling intensity above the mounds is relatively low at neap tide (Figs 3a and 4a) leaving the deep water-column poor in organic matter (Fig. 3E). During spring tide however, up- and downwelling currents intensify (Fig. 3B) resulting in organic matter plumes that are directed from the surface ocean towards the CWC-rich Logachev mounds (Fig. 3F). This localised downwelling occurs repeatedly during the lunar tidal cycle, but strongest downwelling events are observed during spring tide (Supplementary Movie). Towards the end of the lunar tidal cycle, vertical currents weaken and the deep water becomes more oligotrophic again (Supplementary Movie). From these simulations, it is clear that food supply is mainly sustained through vertical downwelling events that establish a direct link between 600-m deep cold-water coral mounds and surface productivity.

Along the shelf break ridge, the CWCs receive organic matter through a different transport mechanism (Fig. 3G,H). Vertical up- and downwelling is also observed above the ridge, but the intensity is lower (Fig. 3C,D) and does not generate directed organic matter plumes as found for the CWC mounds (Fig. 3G,H). Instead, there is retention of organic matter-rich waters above the shallow Rockall Bank that occasionally spills over the ridge down the slope (Fig. 3H). Hence, the dominant transport mechanism of organic matter to the CWCs on the shelf ridge appears to be downslope transport.

### Organic matter distribution and sequestration

The high mineralization rates of CWC reef communities suggests that a local ‘focussing’ of organic matter onto these ecosystems must occur, which potentially lowers the availability of organic matter for the surrounding seafloor^3^. We evaluated this hypothesis quantitatively by plotting the modelled organic matter deposition against water depth across the model domain (Fig. 5A). The organic matter deposition on the CWCs on the mounds and ridge was elevated with a factor 5 to 10 as compared to the off-reef seafloor at similar water depth (Fig. 5A). A comparison of these initial model results with a model simulation in which the filtration activity of CWC was toggled off shows that this separation between organic matter deposition of CWCs versus the off-reef seafloor vanishes (Fig. 5B). Hence, the filtration activity of CWCs results in a ‘focussing’ of organic matter onto the CWCs at the expense of deposition on the off-reef seafloor at comparable water depth.

The interaction of CWC mounds and the shelf ridge supplies food to the CWCs, but this ‘topographically-enhanced pump’ may also enhance the sequestration of organic matter to the deep sea. To isolate the importance of hydrodynamic transport for the organic matter sequestration within the model domain, we ran a simulation in which organic matter was subjected only to passive sinking, biological degradation and CWC filtration activity. Interestingly, the organic carbon deposition beyond 1200 m water depth is up to an order of magnitude lower in the model simulations that lack hydrodynamics (Fig. 5C). This implies that part of the organic matter that is transported downward due to the topographically-enhanced pump ‘leaks’ away and settles on the seafloor at greater depths. Hence, the topographically-enhanced pump acts as a conduit of organic matter supply to the deeper seafloor.

Another striking feature that becomes clear from the model run in which the hydrodynamics were toggled off (Fig. 5C) is that organic matter deposition by mere passive sinking are grossly insufficient to sustain the high mineralization activity of CWC (measured to be between 8 to 88 mmol C m^−2^ d^−1^ 2,3,23,24). While it is well-established that cold-water corals occur at locations with elevated current velocities, internal waves or turbulent mixing^10^,^16^,^17^,^25^,^26^, our model results provide direct evidence that hydrodynamic forcing is essential to sustain the metabolic activity of CWCs in the deep sea.

### Implications and outlook

A fundamental question in oceanography is how CWCs can thrive in the food-limited deep ocean. The dominant view on this paradigm is described by the so-called ‘environmental control theory’^27^, which assumes that environmental factors control the distribution of CWCs. Key elements of this theory are that CWCs are found in regions with a relatively high surface productivity^7^ and enhanced hydrodynamic energy^26^, including high bottom currents^16^, retention within Taylor columns^28^,^29^ and breaking internal waves^16^,^30^. These factors ensure a sufficient supply of surface-derived organic matter to the filter-feeding CWCs^9^,^31^ and explain CWC occurrences in for example submarine canyons^32^ and sills^11^. Abiotic variables such as seawater density^33^, temperature^25^ and aragonite saturation state^34^ may further narrow down the suitable habitat of CWCs. Here we show that the seafloor topography that is created by the CWCs leads to a preferential focussing of organic matter towards these ecological hotspots. This focussing implies a positive feedback between CWC growth and food supply. Hence, rather that being under mere environmental control, CWCs modify their environment through mound formation, whereby food supply is stimulated. This positive feedback is a remarkable example of large-scale and long-term ecosystem engineering (*sensu*^35^). Hence, a more appropriate term to coin the factors that control CWC distribution would be the ‘ecosystem engineer control theory’.

**Figure 1:**
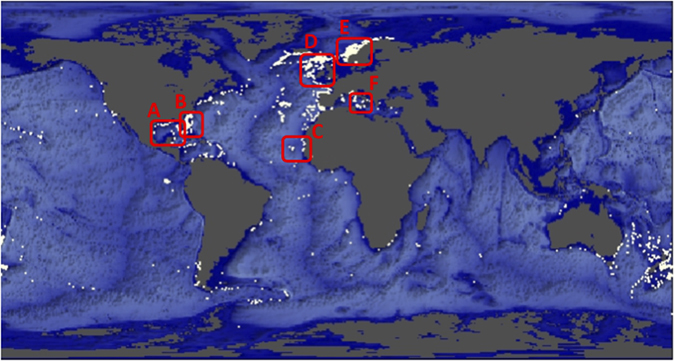
Global map with occurrences (white dots) of Scleractinian cold-water corals^57^ (data available at http://data.unep-wcmc.org/datasets/3). Red boxes indicate known locations along the continental shelves where *Lophelia pertusa* has shaped the seafloor topography into (**A**) a coral province of 20–40 m high in the Gulf of Mexico^58^, (**B**) coral ridges of up to 20 m high in Florida Strait^59^, (**C**) elongated mound clusters with the largest mounds reaching a height of 100 m off the coast of Mauritania^60^, (**D**) numerous and large carbonate mounds of up to 300 m high along the Irish margin^16^, (**E**) elongated CWC reefs, mostly between 10 and 15 m high, all along the Norwegian shelf^3^,^31^ and (**F**) up to 25-m high coral banks in the Mediterranean Sea^61^. Figure and basemap created with the R-package plot3D, version 1.1 https://cran.r-project.org/package=plot3D.

**Figure 2:**
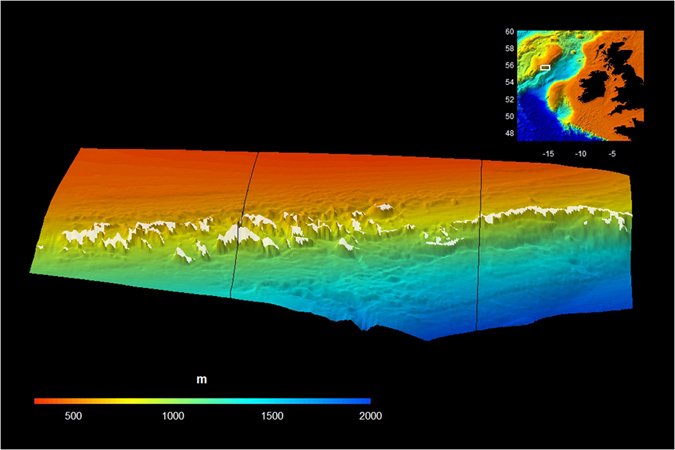
The modelled area of Rockall Bank with the two contrasting CWC growth forms: CWC growth on mounds (transected by the left line) and CWC growth on a ridge (transected by the right line). The dimension of the area is 90 km (longitude) × 60 km (latitude) × 2.4 km (depth). Scale bar indicates depth of the seafloor. White dots indicate the predicted CWC occurrences from the habitat suitability model (see Methods). Inset figure shows the location of Rockall Bank along the Irish margin. Figure created with the R-package plot3D, version 1.1 https://cran.r-project.org/package=plot3D.

**Figure 3:**
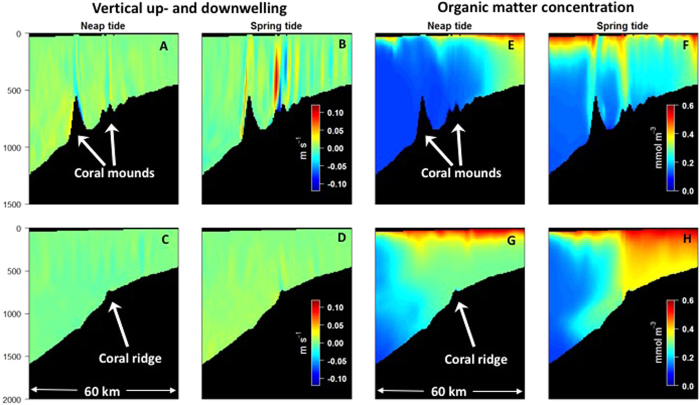
Model output of vertical current velocities (**A–D**) and organic matter concentration in the water column (**E–H**) along the coral mound (**A,B,E,F**) and coral ridge transect (**C,D,G,H**) during neap and spring tide.

**Figure 4:**
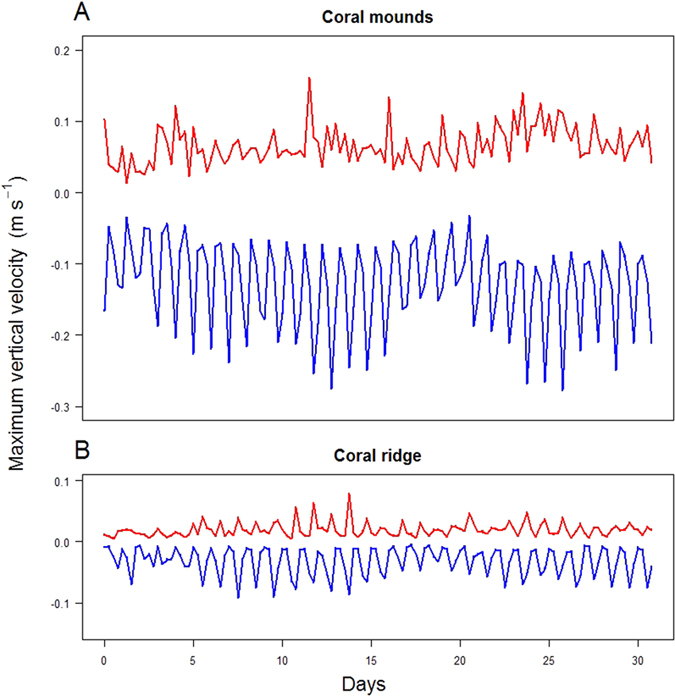
Maximum up- (in red) and downwelling (in blue) current velocities (m s^−1^) occurring simultaneously in the model simulations above the coral mound region (**A**) and coral ridge region (**B**).

It is challenging though to extrapolate from this single case study and assess whether this transport mechanism is important for the CWC reefs around the globe (Fig. 1) or other benthic ecosystems that are associated with seafloor topographies. However, it is well established that topographic rises, of both biogenic and geological origin, are ubiquitously present on the ocean floor^36^, which suggests that organic matter sequestration to the deep sea through a topographically-induced carbon pump could be of global importance. Indeed, predictions of the downward organic matter flux in the water column based on sinking, turbulent mixing and degradation of organic particles, i.e. the canonical Martin formula, often strongly underestimate organic matter deposition on the seafloor, both in localised settings^37^,^38^ as on an ocean-wide scale^39^. While Global Biogeochemical Ocean Circulation Models (GBOCMs) realistically simulate large-scale oceanic circulation^40^, they often tend to underestimate the flux of organic matter to the deep seafloor^41^. Based on our results, part of this discrepancy could result from a too crude parameterisation of the small-scale topographic features within the coarse model grids of GBOCMs and thereby they miss the contribution to the organic matter flux due to topographically-enhanced sequestration.

Our study enhances the mechanistic understanding of the transport of organic matter to CWCs. This knowledge is not only crucially important to understand the functioning of CWCs in the present-day, but also in view of our future oceans, in which the export of organic matter to the deep sea is expected to decrease^6^ due to increased stratification, with major anticipated consequences for soft-sediment communities^42^. It is acknowledged that CWCs experience a multitude of stressors that makes them particularly vulnerable to expected global changes. Increased water-column stratification may not only reduce primary production due to nutrient limitation, but may directly reduce the frequency and magnitude of vertical mixing events decreasing the organic matter supply. Simultaneously, organic matter demands will likely increase, both because CWC strongly increase respiration at elevated temperatures^43^ and because of increased energy demands to sustain calcification rates^44^ in aragonite-undersaturated seawater caused by ocean acidification in the next decades^7^. It is clear that only an integrated approach, involving hydrodynamic, biogeochemical and physiological disciplines, will allow forecasting the functioning of precious CWCs in our future oceans.

## Methods

### Material and methods

The Logachev Mound province is situated on the south-western flank of Rockall Bank (NE Atlantic). Mound clusters are several kilometres long and have an elongated shape that follow the isobaths of Rockall Bank and have their summit between 600 and 1000 m water depth^15^. The summits and rims of these mounds are capped with cold-water corals and their rich associated communities^2^,^16^,^45^. Our conclusions are derived from a coupling of a hydrodynamic, biogeochemical and habitat suitability model, which are described below.

### Hydrodynamic model

A hydrodynamic model of the Logachev mounds has been developed by ref. 17 in the Regional Ocean Modeling System (ROMS), which solves the free-surface hydrostatic primitive equations of the fluid dynamics over complex topography using the Boussinesq assumption^46^,^47^. The finite difference approximations have stretched, terrain-following coordinates in the vertical and curvilinear Arakawa C-grid coordinates in the horizontal. A nested grid was used with a parent domain of 192 * 188 km, and a child domain of 103 * 61 km. The horizontal spatial resolution of the internal grid (55°21′ to 55°53′ North, 16°16′ to 14°50′ W) was high, comprising 410 * 242 grid cells, each about 250 m wide. Along the vertical, there were 32 sigma levels that provided increased resolution near the bottom and surface boundaries^47^. The stretching parameters to generate this grid were *θ*_*s*_ = 3.4, *θ*_*b*_ = 1^17^. The Northern, Eastern, Southern and Western boundary conditions were imposed as an implicit radiative boundary scheme for water-surface elevation and the 3D momentum, allowing waves to propagate out of the domain. The currents were forced by the global inverse tidal solution TPXO6 48; the model was run for an entire year, but only the results for the situation in April was used. It is important to note that although non-linear internal wave generation is not explicitly resolved by the model, the evolution of internal lee waves and associated large-amplitude up- and downwelling events are well represented^17^.

### Habitat suitability model

The presence of cold-water corals in the area was inferred from a habitat suitability model, which predicts coral occurrence at the Logachev province from a generalised linear modelling approach based on a set of bathymetric, hydrodynamic and environmental variables^14^. The hydrodynamical variables were extracted from the ROMS model by^17^ as used in the present study. The model was trained on a coral presence/absence dataset that was collected during dedicated Remotely Operated Vehicle (ROV) transects (±2 m above the seabed, speed 0.3 m/s), providing frames with 117 presences and 227 absences within the Logachev province^14^. The model performed well after training and predicts coral presence primarily on the summit and flanks of the mounds provinces in the south-western Rockall Bank and on a ridge in the north-western Rockall Bank^14^.

### Organic matter reaction model

The output of the hydrodynamic ROMS model, stored at 6 hour intervals, was used offline to drive organic matter dynamics in the water column, including production at the upper boundary, advective horizontal and vertical transport, constant decay and passive sinking. The ROMS model includes the effects of waves on the ocean currents by a moving surface boundary^49^. This temporal stretching of the grid was ignored in the simulation of the organic matter dynamics to shorten simulation time. Conservation of momentum was imposed at each time step by calculating the upward flow rate *w* from the flux divergence of the horizontal flows and assuming that the free-surface elevation (*η*) is constant and that the vertical velocity at the bottom is zero *w*_*z*=0_ = 0


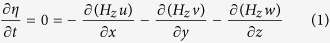


in which *H*_*z*_ is the grid-cell thickness.

The organic matter in the model is expressed in Carbon equivalent (*C*). Following the conservation of momentum, the organic matter reaction model is given by:


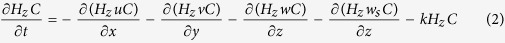


in which *w*_*s*_ is the sinking velocity, and *k* is the first-order decay rate, both taken as a constant.

Zero-gradient conditions were imposed at the lower and horizontal domain boundaries. At the upper boundary a constant flux of 12 mmol C m^−2^ d^−1^ was imposed. This is equivalent to an export carbon flux of about 50 g C m^−2^ yr^−1^, consistent with estimates of new production in the nearby Goban Spur area^50^. The sinking velocity (*w*_*s*_) and the decay rate (*k*) were chosen such that the e-folding depth (*w*/*k*) was around 500–700 m, a value often used in biogeochemical ocean general circulation models^39^. The sinking velocity of 20 m d^−1^ and the first-order decay rate of 0.03 d^−1^ are representative for the decay of freshly produced organic matter^51^. Note that with this input flux and sinking speed, the maximal organic matter concentration in the water column is 0.6 mmol C m^−3^. This is below measured organic matter concentrations at northwestern Atlantic margins, ranging from 0.75 mmol C m^−3^ at Darwin Mounds to 3.7 mmol C m^−3^ at Logachev mounds and up to 10.8 mmol C m^−3^ at Porcupine Bank^52^. However, our model results are representative for reactive, freshly-produced organic matter, while the *in situ* measurements include refractory organic matter.

**Figure 5:**
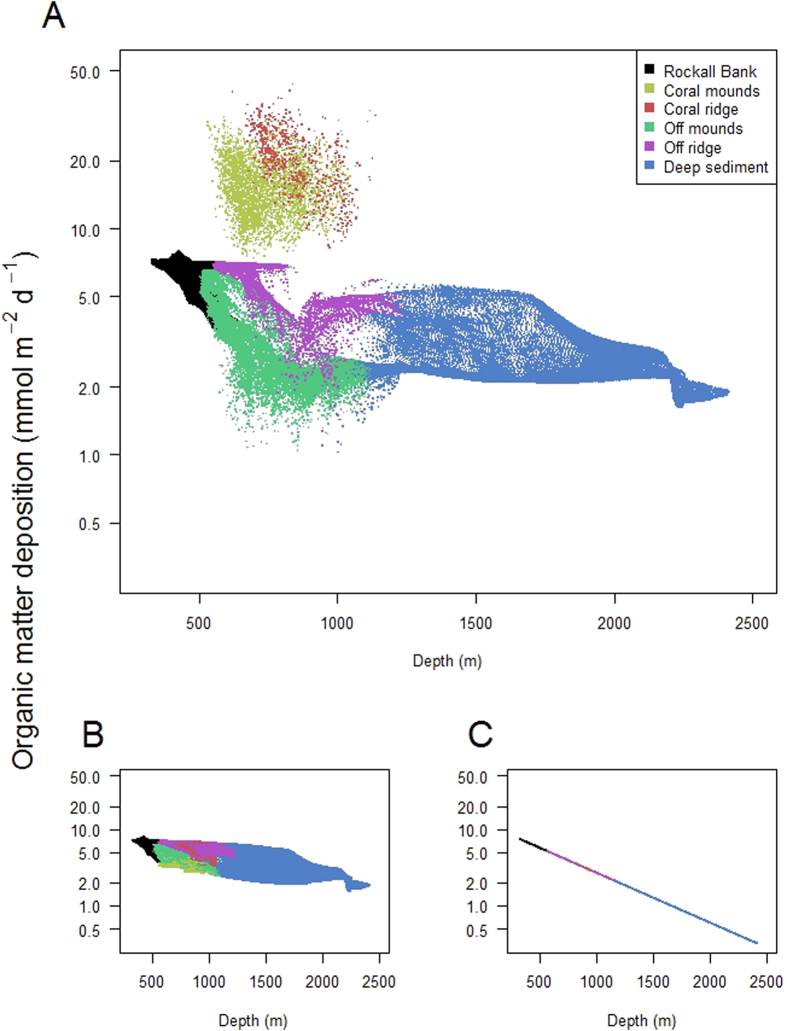
(**A**) Modelled organic matter deposition versus water depth in the various habitats within the model domain, (**B**) organic matter deposition versus water depth in absence of CWC filtration activity and (**C**) organic matter deposition versus water depth in the absence of hydrodynamics.

Deposition of organic matter from the water column to the seafloor is due to passive sinking. In the CWC areas however, the deposition of organic matter is due to both passive sinking and suspension-feeding. Feeding by passive (e.g. corals, anemones and soft-corals) and active (e.g. bivalves and sponges) suspension feeders is often expressed in units of m^3^ m^−2^ d^−1^ = m d^−1^, which, when multiplied with the OM concentration in the water column, gives total OM uptake rates^53^. This expression is mathematically similar to the expression for passive sinking, so that suspension feeding can be accounted for by increasing the passive settling velocity (*w*_*s*_). The passive sinking velocity in the bottom grid cells overlying the CWCs was enhanced by a factor 10 to account for the suspension feeding activity, giving a *w*_*s*_ of 200 m d^−1^. Note that the organic matter deposition presented in Fig. 5A can be easily back-calculated into ‘passive settling’ and ‘suspension feeding’ using this enhancement factor. The enhancement factor of 10 can be justified by the following three arguments. Firstly, the metabolic activity of a cold-water coral community is 5 to 10 times higher as compared to the adjacent barren seafloor^2^,^3^,^23^,^24^. The enhancement factor 10 is needed to ensure that sufficient organic matter was taken up by the reef community to sustain their elevated metabolic activity. Secondly, while *in situ* estimates of *w*_*s*_ above CWC reefs are not available, our enhancement factor compares favourably with data from tropical coral reefs^53^. These authors estimated grazing rates (conceptually comparable to *w*_*s*_) for tropical reefs of −60 (when reefs are a net source of organic matter) up to 180 m d^−1^. As CWC reefs cannot be net sources of organic matter and the friction velocities are typically higher than on tropical reefs^54^, the higher values for *w*_*s*_ from^53^ are more reasonable for CWC reefs. Thirdly, an order of magnitude calculation of the capturing efficiency shows that the assumed *w*_*s*_ is not unrealistic. The mean current velocity is roughly 0.3 m s^−1^ above the CWCs. Assuming that suspension feeders feed from 20 cm of water above the sediment (a lower estimate, as corals are usually higher than 20 cm), this means that a m^2^ of CWC reef can extract resources from 5184 m^3^ d^−1^. With the imposed suspension feeding rate of 200 m d^−1^, this implies that they have a capturing efficiency of less than 4%.

The biogeochemical transport-reaction equations were numerically solved on a staggered grid (Arakawa C-grid) with the organic matter concentration defined in the centre of each box and the flows on the grid cell interfaces. The six-hourly velocity data from the hydrodynamic model were linearly interpolated in time to obtain velocity fields at each model integration step. The organic matter model comprised about 2.5 · 10^6^ equations and was numerically integrated using a variable-order Adams-Moulton predictor-corrector scheme, as implemented in the R-package deSolve^55^ running in the open-source software R^56^. Advection was implemented using simple first-order upwind differencing; due to the numerical dispersion that this generates, no horizontal or vertical dispersion was used. The vertical diffusivities as generated by the hydrodynamic model were in fact very low.

## Additional Information

**How to cite this article**: Soetaert, K. *et al*. Ecosystem engineering creates a direct nutritional link between 600-m deep cold-water coral mounds and surface productivity. *Sci. Rep.* **6**, 35057; doi: 10.1038/srep35057 (2016).

## Supplementary Material

Supplementary Information

Supplementary Information
