# Supplementary material for: Ecosystem engineering creates a direct nutritional link between 600-m deep cold-water coral mounds and surface productivity
Source: Sci Rep. 2016 Oct 11;6:35057. doi: 10.1038/srep35057 (PMC5057138; doi:10.1038/srep35057)
Supplement: Supplementary Information [file srep35057-s1.pdf]

Ecosystem engineering creates a direct nutritional link between 600-m deep cold-water coral mounds and surface productivity

Karline Soetaert,Christian Mohn, Anna Rengstorf, Anthony Grehan and Dick van Oevelen.

Supplementary movie

Vertical current velocities (left) and organic matter concentrations (right) during the entire simulation period at 6 hour resolution, for the coral mound region (upper half) and the coral ridge region (lower half).

Supplementary figure

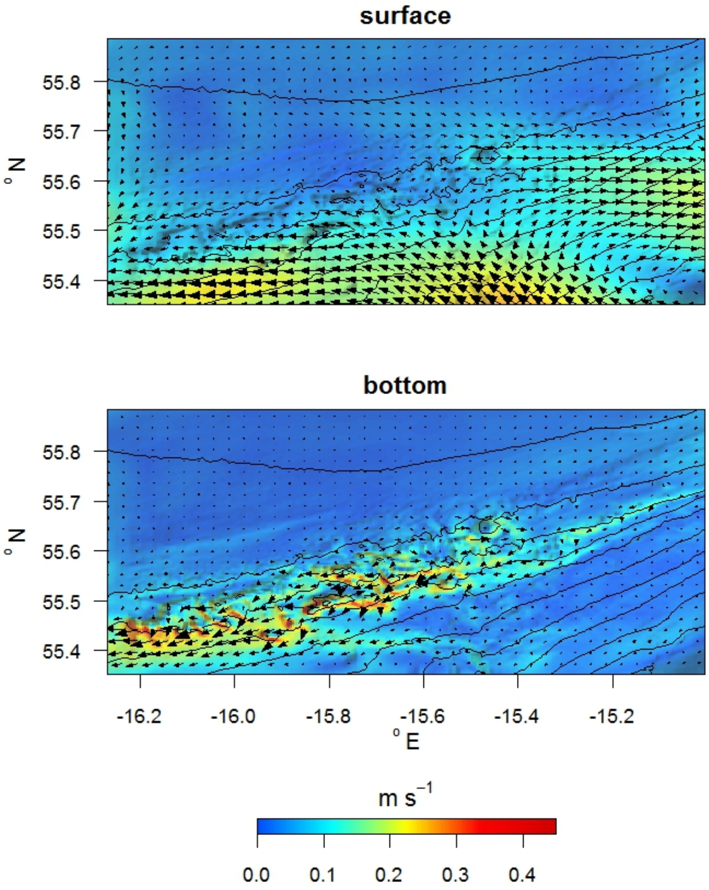

Figure 1: Residual currents in the eastern Rockall Bank (NE Atlantic Ocean), calculated from the hydrodynamic model output, in the surface water (upper) and near the bottom (below)
